# Sustainable Silk Fibroin Nanofibers Membranes with Natural Photothermal and Bioactive Components for Adhesive-Free Soft Tissue Repair

**DOI:** 10.1021/acsabm.5c02042

**Published:** 2026-01-23

**Authors:** Martina Corsini, Livia Ottaviano, Luana Mariani, Marianna Barbalinardo, Giada Magni, Francesca Rossi, Fulvio Ratto, Anna Donnadio, Roberto Zamboni, Annalisa Aluigi, Giovanna Sotgiu, Tamara Posati

**Affiliations:** † Consiglio Nazionale delle Ricerche, 201838 Istituto per la Sintesi Organica e la Fotoreattività (CNR-ISOF), via Piero Gobetti 101, 40129 Bologna, Italy; ‡ Consiglio Nazionale delle Ricerche, Istituto per lo Studio dei Materiali Nanostrutturati (CNR-ISMN), via Piero Gobetti 101, 40129 Bologna, Italy; § Consiglio Nazionale delle Ricerche Istituto di Fisica Applicata Nello Carrara (CNR-IFAC), Via Madonna del Piano 10, 50019 Sesto Fiorentino, Italy; ∥ Dipartimento di Scienze Farmaceutiche, Università di Perugia, Via del Liceo, 1, 06123 Perugia, Italy; ⊥ Dipartimento di Scienze Biomolecolari, Università di Urbino, Piazza del Rinascimento 6, 61029 Urbino, Italy

**Keywords:** silk fibroin, nanofibers, cuttlefish ink, vitamin B2, adhesive free tissue repair

## Abstract

Multifunctional silk fibroin (SF) nanofibrous membranes incorporating cuttlefish ink (CI) and vitamin B2 (VitB2) were developed via water-based electrospinning as bioactive scaffolds for soft tissue regeneration and laser-assisted tissue welding. CI provided antioxidant and photothermal properties, while VitB2 promoted cellular proliferation. Membranes exhibited controlled VitB2 release and CI retention within the matrix. NIH-3T3 fibroblast assays confirmed high viability (>80% at 48 h), with CI promoting adhesion and cytoskeletal organization, and VitB2 enabling formation of a confluent, organized cell layer. Laser-assisted welding produced satisfactory adhesion and shear-stress resistance on the order of ten kPa in ex vivo tendons, corneas, and sclerae, while keeping tissue temperatures below the 60 °C threshold for thermal damage. These findings highlight CI- and VitB2-loaded SF membranes as sustainable, cytocompatible scaffolds with antioxidant and adhesive functionalities, offering a versatile platform for soft tissue repair via an adhesive-free approach.

## Graphical abstract

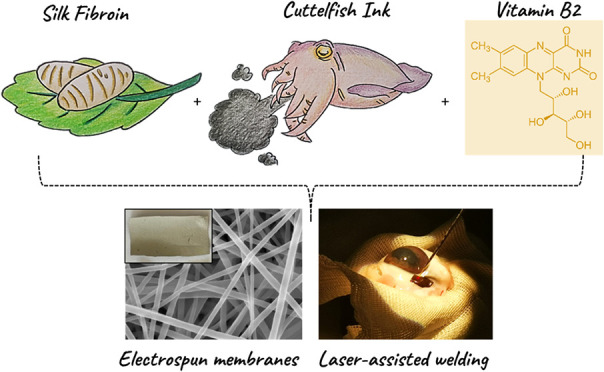

## Introduction

The development of advanced biomaterials for tissue engineering and regenerative medicine has led to significant breakthroughs in minimally invasive surgical techniques, such as tissue welding. In contrast to conventional methods such as chemical adhesives, suturing, or stapling, light-activated tissue bonding presents a viable alternative that mitigates tissue trauma, promotes favorable healing outcomes, and enhances precision in delicate surgical procedures. This technique relies on photosensitizers and light to induce cross-linking at the tissue interface via photochemical or photothermal effects, enabling rapid, localized, and strong adhesion that is crucial for soft tissue repair.^1–3^ Silk fibroin (SF), a fibrous protein derived from *Bombyx mori* cocoons, has garnered significant attention in tissue engineering due to its excellent biocompatibility, biodegradability, tunable mechanical properties, and ability to form nanofibrous scaffolds.^4–6^ These scaffolds closely mimic the extracellular matrix, making them highly suitable for applications in tissue repair, guided tissue regeneration, and drug delivery.^7–9^ Furthermore, electrospun SF membranes provide an effective platform for promoting cell attachment and proliferation, making them valuable in soft tissue repair, including tendon and corneal tissue regeneration.^10,11^

Riboflavin (vitamin B2, VitB2), a water-soluble vitamin, plays an essential role in cellular metabolism and energy production, particularly through its function as a precursor to flavin mononucleotide (FMN) and flavin adenine dinucleotide (FAD). These cofactors are integral to numerous redox reactions, regulating mitochondrial function and cellular energy metabolism, and indirectly contributing to antioxidant defense by supporting the activity of enzymes such as glutathione reductase.^12,13^ Beyond its metabolic role, VitB2 has gained attention for its photochemical activity, particularly in corneal cross-linking, where it has been extensively used in ophthalmic applications to enhance the stability and integrity of corneal tissue.^14–16^ Additionally, riboflavin has been shown to promote regeneration in soft tissues, such as tendons, by facilitating wound healing, mitigating oxidative damage, and supporting cellular proliferation in response to injury.^17,18^ These properties make riboflavin a promising candidate for incorporation into bioactive scaffolds aimed at soft tissue repair, representing a step beyond conventional VitB2 applications.

Cuttlefish ink (CI), composed mainly of melanin and proteins, offers unique photothermal properties, antioxidant activity, and light-absorbing capabilities. Melanin, a key component of CI, has demonstrated significant potential in enhancing tissue welding by promoting localized heating when exposed to light, thus accelerating the adhesion of tissues at the injury site.^19,20^ This photothermal effect, combined with its antioxidant properties,^21–23^ makes CI a further attractive additive for bioactive scaffolds designed for tissue repair and regeneration. Despite progress with synthetic photothermal systems such as polydopamine, carbon nanotubes, and metallic nanoparticles,^24–28^ challenges remain for clinical translation, including potential cytotoxicity, complex synthesis, and limited biocompatibility. In this context, CI, as an optical contrast agent, represents a fully biobased and minimally processed source of melanin that can be readily integrated into silk fibroin scaffolds, providing stable photothermal properties and high biocompatibility. In combination with riboflavin, CI enables complementary photochemical and photothermal interactions, creating a dual light-activated mechanism within a single scaffold. This multifunctional design introduces a novel approach to light-activated tissue bonding and bioactive scaffold development, distinct from systems that rely solely on photothermal agents or silk-based materials.

The aim of this work is therefore to develop a multifunctional electrospun membrane based on silk fibroin, incorporating VitB2 and CI, designed for developing bioactive scaffolds capable of supporting soft tissue regeneration and laser-assisted tissue welding. Membranes were characterized morphologically and structurally via scanning electron microscopy (SEM) and ATR-FTIR spectroscopy, respectively, and then evaluated for VitB2 release kinetics in physiological buffer (PBS, pH 7.4), and enzymatic stability. Laser welding experiments were performed on *ex vivo* rabbit tendons, as well as ovine corneal and scleral tissues, using an 810 nm diode laser, followed by mechanical testing to assess weld integrity. *In vitro* biological assays were performed to evaluate the biocompatibility of the developed scaffolds with mouse embryonic fibroblasts. Collectively, this study proposes a sustainable and biocompatible platform for adhesive-free tissue repair, with potential applications in soft connective tissues and ophthalmic surgery, bridging the fields of photomedicine, biomaterials, and regenerative engineering. The multifunctional design aims to deliver mechanical support, photothermal enhancement, controlled drug release and oxidative stress protection within a single bioengineered membrane.

## Results

### Extraction and Characterization of Cuttlefish Ink (CI)

Cuttlefish Ink was directly collected by emptying the ink sacs of *Sepia officinalis*. The subsequent purification phase is crucial for removing mucus and lipid substances, resulting in a purified ink mainly composed of melanin.^29^ The properly purified CI was then analyzed via scanning electron microscopy (SEM). As shown in Figure 1B, CI consists of nanoparticles with an average diameter of 123 ± 30 nm, consistent with literature data.^30^ Prior to purification, the nanoparticles show increased aggregation and, in some regions, evidence of partial fusion, which may be attributed to the presence of mucinous or lipid substances (Figure 1A).

**Figure 1:**
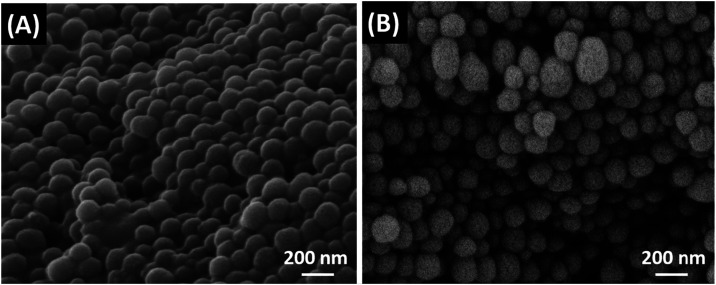
SEM images of CI nanoparticles before (A) and after purification (B).

The antioxidant and radical-scavenging activity of CI was quantified through its reaction with DPPH. As shown in Figure 2A, CI exhibits high antioxidant activity, with an EC50 of approximately 50 μg/mL, representing the concentration required to reduce the DPPH radical by 50%. This value is comparable to that of other well-known antioxidant compounds.^31,32^

**Figure 2:**
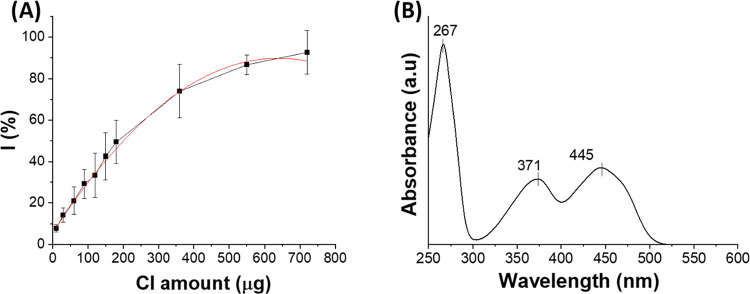
Radical scavenging activity of CI performed with DPPH method (A) and UV–vis absorption spectrum of VitB2 (B).

Vitamin B2 was characterized by UV–vis analysis. The absorption spectrum of VitB2 (Figure 2B), in the range of 250–600 nm, exhibits the typical three bands with maxima at 267, 371, and 445 nm as reported in literature.^33^

### Preparation of Electrospun Membranes Based on SF, PEO, CI, and VitB2

The nanofibrous membranes were prepared by adding glycerol (20% w/w vs SF) to the SF solution, which conferred water insolubility to the spun membranes. Additionally, PEO (at a 70:30 ratio of PEO to SF; total polymer concentration of 5% w/v) was included as a supporting polymer to increase the viscosity of the fibroin solution for successful electrospinning. As shown in Figure 3, the electrospun nanofibrous membranes were flexible and self-sustaining, with a thickness of approximately 100 ± 20 μm, obtained with 1 mL of solution.

**Figure 3:**
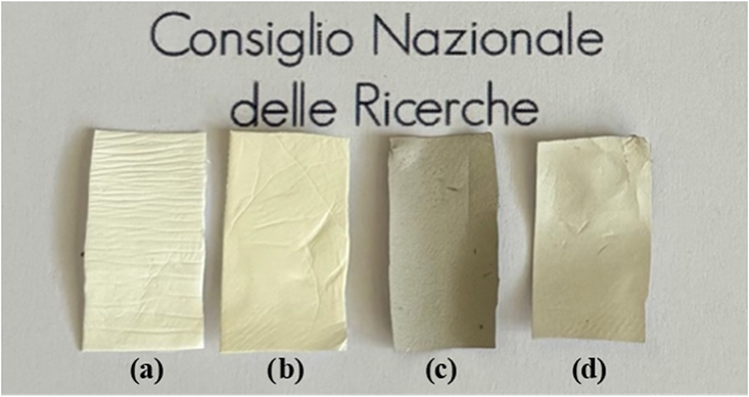
Visual appearance of electrospun membranes: (a) SF-Pgl; (b) SF-Pgl/VitB2; (c) SF-Pgl/CI; (d) SF-Pgl/CI+VitB2.

The obtained fibers were morphologically characterized by SEM. As shown in Figure 4, all samples display randomly oriented nanofibers forming a nonwoven fabric, with negligible defect levels. The average fiber diameters, calculated over 100 nanofibers (Figure SI 1), showed a slight increase upon incorporation of either VitB2 or CI, rising from 160 nm for SF-Pgl to 178 and 184 nm for SF-Pgl/VitB2 and SF-Pgl/CI, respectively. Notably, the presence of CI also resulted in a broader size distribution, indicating decreased uniformity in fiber morphology. Interestingly, when both VitB2 and CI were combined, the resulting fiber diameters were comparable to those of the control sample (161 nm), suggesting a compensatory interplay between the two additives.

**Figure 4:**
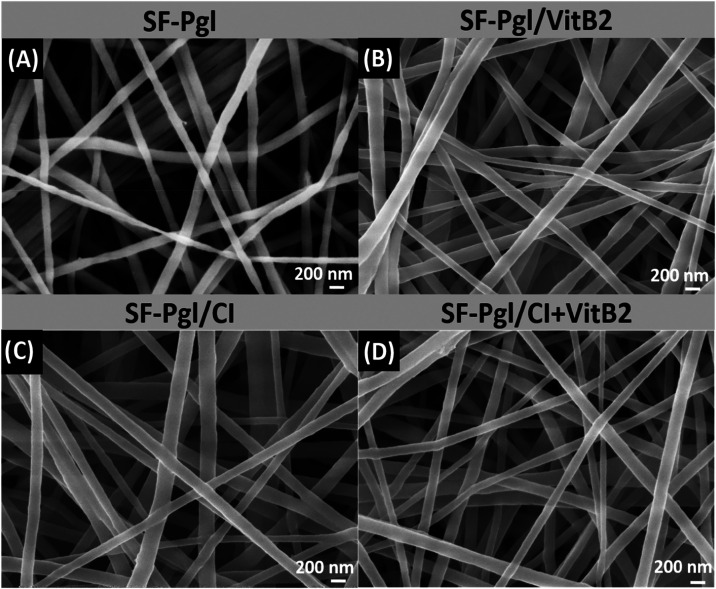
SEM images of (A) SF-Pgl, (B) SF-Pgl/VitB2, (C) SF-Pgl/CI and (D) SF-Pgl/CI+VitB2.

To evaluate the SF structure in electrospun membranes and potential changes induced by CI and VitB2, Fourier-transform infrared spectroscopy in attenuated total reflectance mode (FT-IR/ATR) was employed. In proteins, IR absorption bands in the 1400–1800 cm^–1^ spectral region primarily arise from vibrational modes of the peptide amide group and can thus be correlated with the secondary structure of the protein. The peptide group gives rise to characteristic bands: the amide I band (1600–1700 cm^–1^), mainly due to C = O stretching, and the amide II band (1500–1600 cm^–1^), associated with NH bending and CN stretching vibrations. For SF, bands around 1550 and 1650 cm^–1^ correspond to α-helix and random coil structures (water-soluble Silk I conformation), while bands at approximately 1515, 1620, and 1700 cm^–1^ are associated with β-sheet structures (water-insoluble Silk II conformation).^34,35^ Figure 5A presents ATR spectra of all obtained membranes. The spectra appear similar across all samples, with observable bands at 1515, 1620, and 1700 cm^–1^, indicative of crystalline, water-insoluble β-sheet structures of fibroin. As previously observed in SF-based materials such as sponges and films,^36,37^ this is likely due to the presence of glycerol, which induces insolubility in the membranes. The intensities of the bands at 1650 and 1545 cm^–1^ decrease upon addition of CI and VitB2, either individually or in combination, likely reflecting a reduction in amorphous α-helix and random coil structures. In order to quantify the influence of CI and VitB2 on the supermolecular rearrangements of fibroin macromolecules, the deconvolution of the Amide I and Amide II bands was performed (Table S1). As observed, all samples showed a significant reduction in the bands area corresponding to the α-helix/random coil structures (1545 and 1650 cm^–1^). The reduction observed upon adding CI is comparable to that observed with VitB2. However, it should be emphasized that the effect attributed to VitB2 could be underestimated, as the calculated band area may be affected by the VitB2 absorption bands at 1545 and 1650 cm^–1^.

**Figure 5:**
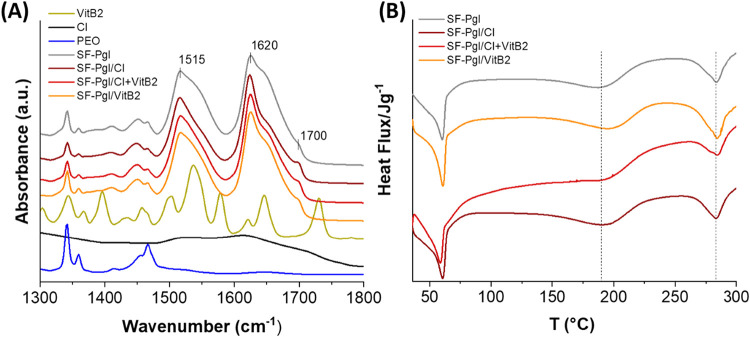
(A) ATR-FTIR spectra in the 1300–1800 cm^–1^ range, shown as vertically stacked plots for clarity. The spectra were normalized to their maximum intensity, and a consistent vertical offset was applied to facilitate comparison among the samples. Key absorption bands are indicated; (B) DSC curves of SF-Pgl, SF-Pgl/VitB2, SF-Pgl/CI and SF-Pgl/CI+VitB2.

To understand the influence of CI and VitB2 on SF crystallization, the thermal behaviors of the membranes were studied using differential scanning calorimetry (DSC). Figure 5B shows that all samples exhibit an endothermic peak around 60 °C, corresponding to PEO melting, and a second endothermic peak at approximately 190 °C, attributed to the transition from unstable noncrystalline structures to β-sheets. In samples containing CI and VitB2, this peak shifts to higher temperatures (between ∼194 and 197 °C, see Table 1), indicating greater protein stability and crystallinity, confirming ATR findings.

**Table 1:** Emperature Values of the Electrospun Membranes

**sample**	Δ ***H*** (J g^**‑1**^ **)**	***T*_m_ (°C)**
**SF-Pgl**	68.40	191.09
**SF-Pgl/VitB2**	64.59	196.81
**SF-Pgl/CI**	72.41	194.07
**SF-Pgl/CI+VitB2**	58.66	194.15

### Mechanical Characterization of the Membranes

Mechanical characterization of the electrospun membranes demonstrated tensile properties consistent with those typically reported for silk fibroin-based mats without additional stiffening treatments^38–41^ (Figure 6). Membranes incorporating cuttlefish ink exhibited a maximum tensile strength of (2.6 ± 0.2) MPa and a Young’s modulus of (18 ± 3) MPa. These values indicate that the inclusion of melanin particles, despite acting as structural heterogeneities, did not compromise the mechanical integrity of the mats. Control membranes without CI showed comparable performance, with a maximum tensile strength of (2.5 ± 0.2) MPa and a Young’s modulus of (18 ± 6) MPa, with no statistically significant differences. Furthermore, the CI-containing samples did not exhibit increased variability, mitigating concerns regarding potential mechanical inconsistency due to particle distribution within the matrix.

**Figure 6:**
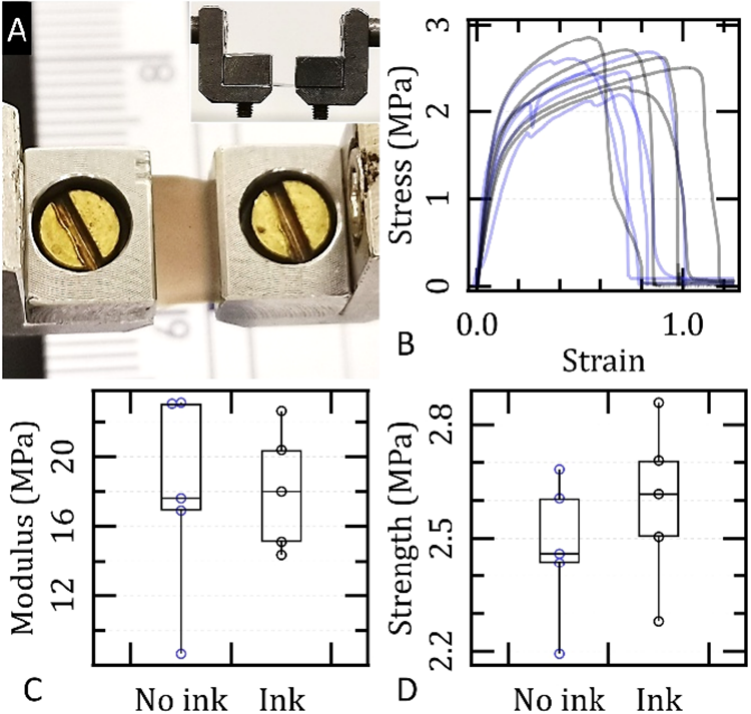
(A) Photograph of the experimental setup used to measure Young’s modulus and maximum tensile strength of the electrospun mats. A lateral view of the system is shown in the inset. (B) Stress–strain curves for SF-Pgl/CI+VitB2 membranes (black curves, *n* = 5) and reference SF-Pgl/VitB2 membranes (blue curves, *n* = 5). (C, D) Box plots of Young’s moduli and maximum tensile strengths derived from the analysis of panel B.

### Stability and Biodegradability of the Membranes

The chemical stability of biomaterials in biological fluids is a crucial requirement in tissue engineering. To evaluate this aspect, membranes were immersed in PBS at 37 °C for 1 day, and the percentage mass loss over time was calculated. Figure 7A shows the weight loss of the SF-Pgl sample used as a reference. All samples exhibited an initial weight loss of approximately 30%, likely due to the release of glycerol and PEO solubility. ATR analysis of the released solutions, analyzed after lyophilization, confirmed the absence of fibroin, indicating its stability in PBS (Figure 7B). This is consistent with the previous ATR spectra of untreated membranes (Figure 5A), which showed a prevalence of β-sheet structures insoluble in water. The stability of the electrospun membranes was further confirmed by SEM analysis. As shown in Figure 7C, the SF-Pgl fibers retained their overall morphology after 24 h of immersion in PBS at 37 °C. However, a noticeable swelling of the fibers and the appearance of small surface pores were observed (fiber diameter 380 ± 100 nm). Quantitative swelling analysis revealed that the fibers exhibited a swelling ratio of 3.9 ± 1.2 for the plain SF-Pgl fibers and 6.2 ± 0.6 for the SF-Pgl fibers containing CI, measured after 24 h. These morphological changes can be attributed to the hydrophilic nature of the components, particularly the water uptake facilitated by glycerol and CI. In addition, the partial leaching of the water-soluble PEO likely contributed to the formation of pores and the slight loosening of the fiber structure. The stability of the membranes was also assessed in the presence of proteases (Figure 7D). The degradation profiles of the various membranes were comparable, with an initial mass loss of 25–35%, reaching approximately 55% within 6 h and stabilizing over 48 h, ultimately leading to a final mass loss of 75–80%.

**Figure 7:**
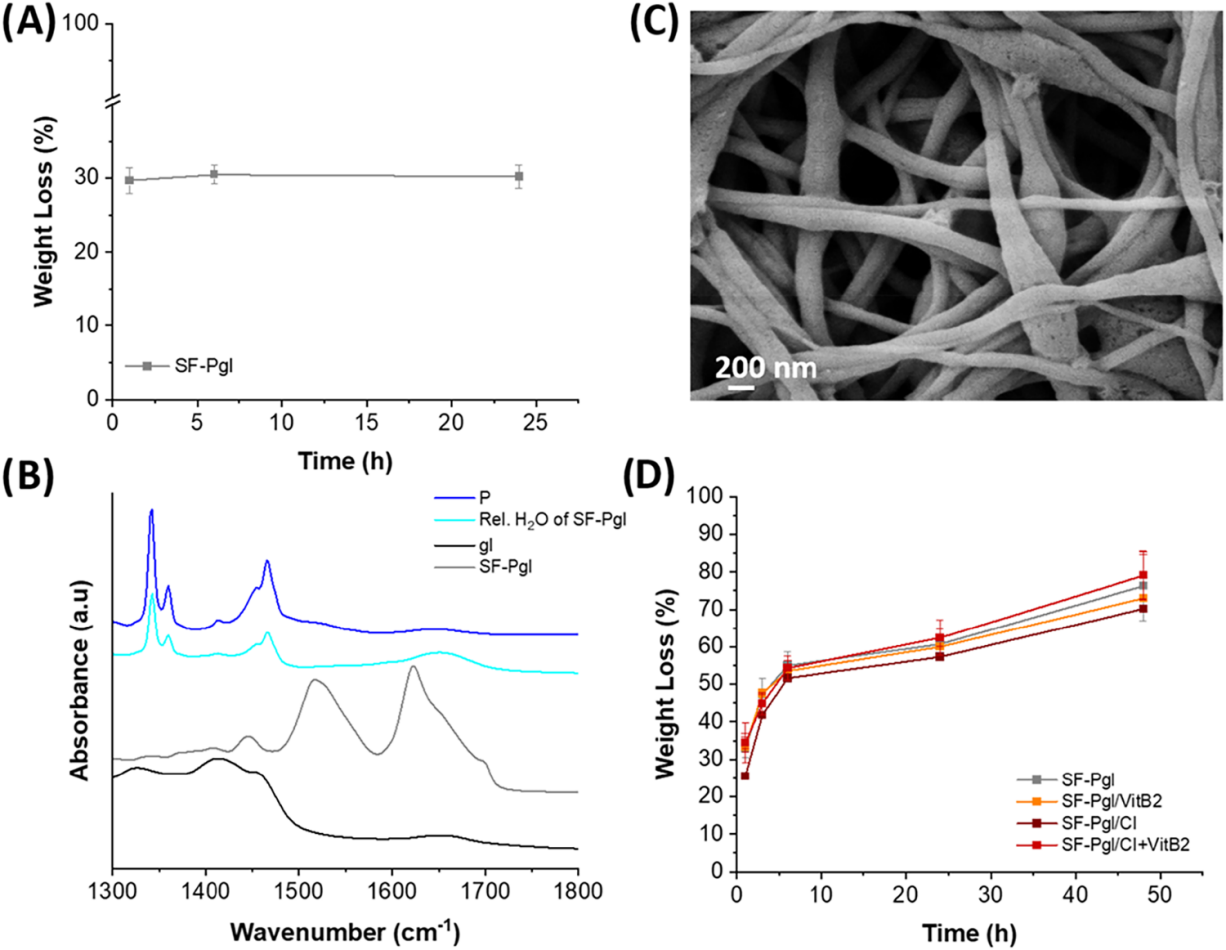
(A) Weight loss percentage in PBS of the SF-Pgl membrane used as a reference over 24 h; (B) ATR spectra of released solutions, shown as vertically stacked plots for clarity. The spectra were normalized to their maximum intensity, and a consistent vertical offset was applied to facilitate comparison among the samples; (C) SEM images of SF-Pgl after 24 h of immersion in PBS pH 7.4; (D) Weight loss percentage of membranes exposed to protease over 48 h.

### Vitamin B2 Release from Electrospun Membranes

VitB2 release from the membranes was evaluated in phosphate-buffered saline (PBS, pH 7.4) at 37 °C. The release was monitored spectrophotometrically by measuring absorbance at 445 nm. No release of CI was detected, as the release solutions at various time points exhibited no antioxidant activity.

To further verify the retention of CI within the fibers, UV–vis spectra of the release media of membranes were collected and reported in the Supporting Information (Figure SI 2). While VitB2 displayed its characteristic absorption peaks, no detectable signal corresponding to CI was observed. Considering that CI consists of nanosized particles that typically generate a broad and intense absorption/scattering band across the UV–vis range, its absence in the release medium strongly confirms that it remained fully confined within the fiber matrix. As shown in Figure 8, the VitB2 release profiles of membranes with and without CI were comparable, indicating that CI exerts minimal influence on release kinetics. In both cases, an initial burst release occurred within the first 30 min, accounting for approximately 50% of total VitB2 release. Subsequently, the release rate decreased, reaching about 80% after 6 h.

**Figure 8:**
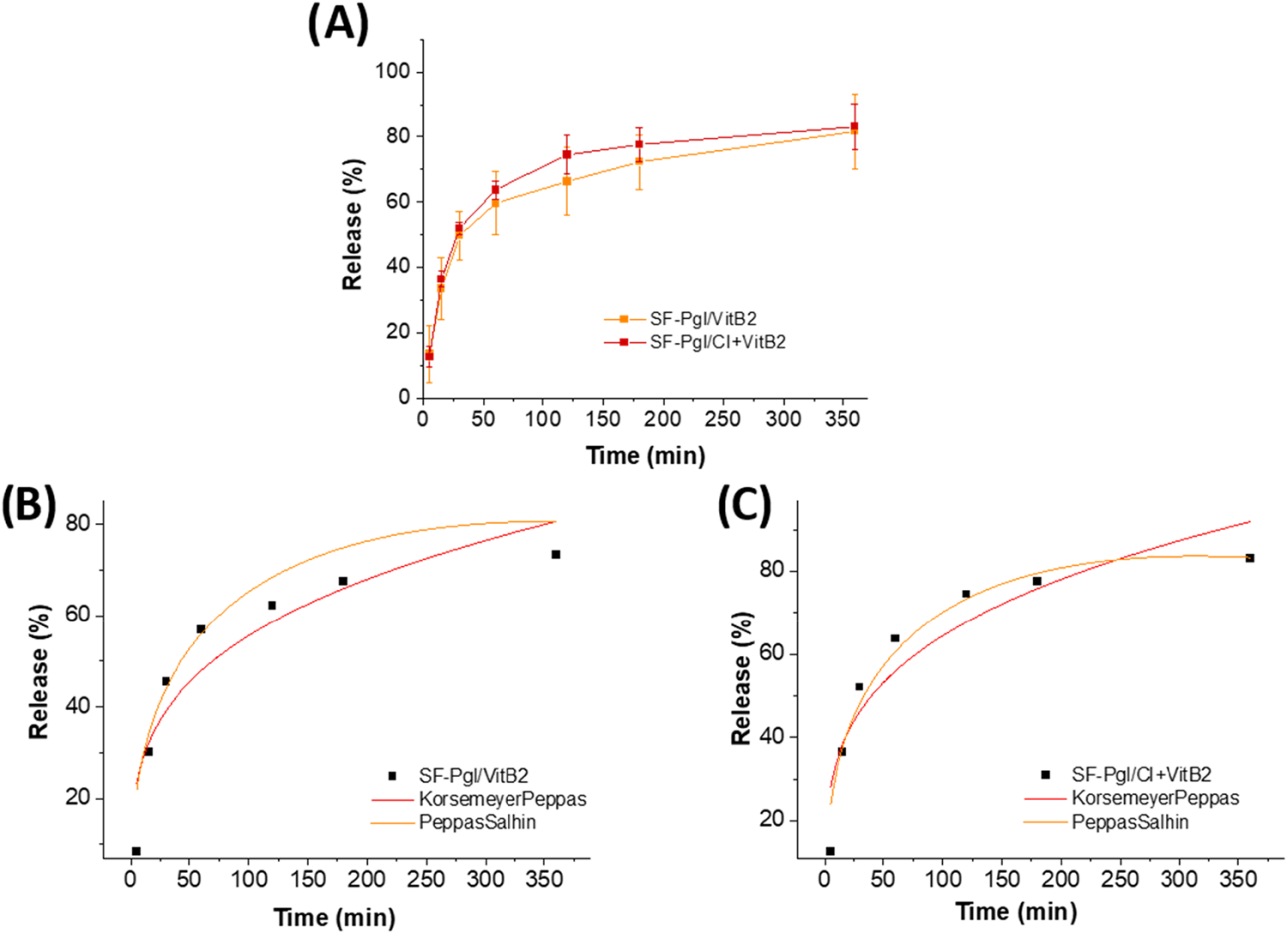
Release kinetics of VitB2 from electrospun membranes: (A) experimental (raw) release data; (B) fitting of the release profile for SF-PgI/VitB2 membranes; (C) fitting of the release profile for SF-PgI/CI+VitB2 membranes.

The *in vitro* release kinetics were analyzed using the Korsmeyer–Peppas and Peppas–Sahlin semiempirical models. The Korsmeyer–Peppas model describes simple diffusion mechanisms, where the release exponent (n) characterizes different transport phenomena: (i) *n* < 0.5, Fickian diffusion; (ii) 0.5 < *n* < 1, anomalous transport (diffusion and swelling); (iii) *n* ≥ 1, case II transport (polymer relaxation-dominated). When the mechanism is not purely diffusive, the Peppas–Sahlin model provides two kinetic constants, *k*_1_ (diffusion contribution) and *k*_2_ (swelling contribution). As reported in Table 2, the Peppas–Sahlin model showed superior *R* ^2^ values (>0.90) for both membrane types. Furthermore, both samples exhibited *n* values <0.5 and negative *k*_2_ values, confirming that VitB2 release follows a Fickian diffusion mechanism.

**Table 2:** Parameters and Correlation Coefficients for the Korsmeyer-Peppas and Peppas-Sahlin Models

	**Korsmeyer-Peppas** ***Q***_***t***_ ***= K***_**KP*t***_^***n***^			**Peppas-Sahlin** ***Q***_***t***_ ***= k***_**1** ***t***_^**0.5**^ ***+ k***_**2** ***t***_^**1.0**^			
**sample**	*K*_KP_	*n*	*R* ^2^	*k*_1_	*k*_2_	*R* ^2^	*|k*1*|*/*|k*2*|*
**SF-Pgl/VitB2**	15 ± 4	0.29 ± 0.06	0.870	11 ± 1	–0.39 ± 0.09	0.939	28
**SF-Pgl/CI+VitB2**	18 ± 5	0.28 ± 0.05	0.880	13 ± 1	–0.47 ± 0.08	0.958	27

Notably, the kinetic constants *K*_kp_ and *k*_1_ were slightly higher for the SF-Pgl/CI+VitB2 membranes compared to SF-Pgl/VitB2 membranes, indicating a moderately faster VitB2 release in the presence of CI. This phenomenon is likely attributable to a weakening of fibroin–VitB2 interactions induced by CI, promoting a more rapid diffusion of the vitamin.

Our preliminary investigation into VitB2 release within rabbit tendons indicated a considerably slower kinetic profile, with a visible signal appearing only after several hours (Figure SI 3). This protracted release is hypothesized to be influenced by factors such as the quality of contact at the interface and its hydration state, aspects that will be rigorously explored in a forthcoming dedicated study.

### Biocompatibility of the Membranes

The biocompatibility of the electrospun membranes was evaluated using the Resazurin Reduction Assay, as detailed in the Experimental section. This assay assessed the viability of NIH-3T3 fibroblasts following 24 and 48 h of exposure to SF-Pgl, SF-Pgl/VitB2, SF-Pgl/CI, and SF-Pgl/CI+VitB2 membranes. Biocompatibility testing is crucial for investigating cellular responses to biomaterials intended for regenerative medicine applications, providing insights into fibroblast survival and metabolic activity upon contact with the substrates. As shown in Figure 9E, all membranes supported cell viability above 90% after 24 h of incubation. After 48 h, cell viability remained higher than the control (SF-Pgl) for all membranes, except for those containing VitB2. Although the presence of VitB2 slightly reduced cell viability, it did not adversely affect the overall biocompatibility, which consistently remained above 80%.

**Figure 9:**
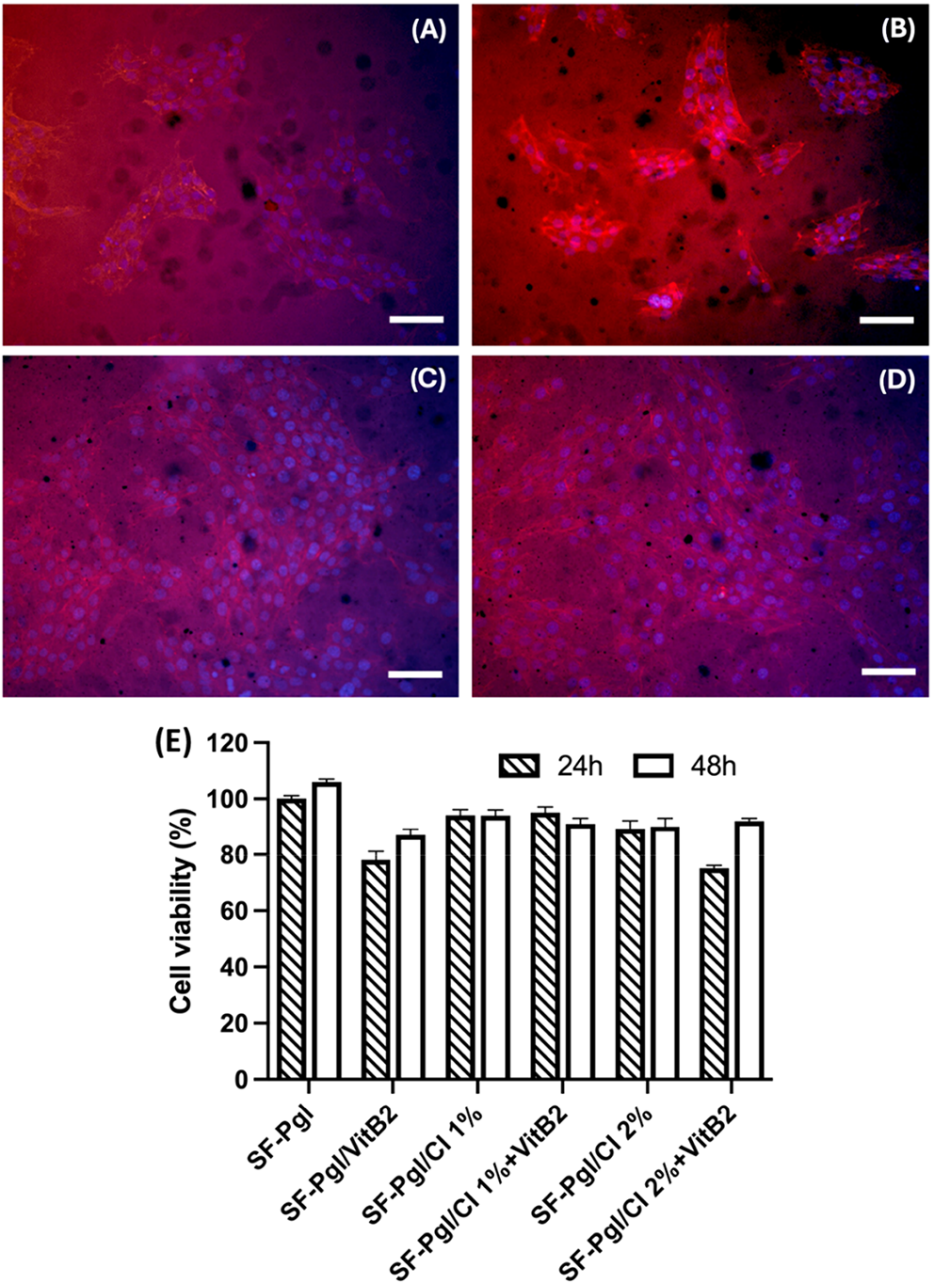
Fluorescence microscopy images of fibroblasts (NIH-3T3) cultured on (A) SF-Pgl, (B) SF-Pgl/CI, (C) SF-Pgl/VitB2, and (D) SF-Pgl/CI+VitB2. Cells were stained for F-actin (red) using phalloidin-TRITC and nuclei (blue) using DAPI. Scale bar: 50 μm. (E) Biocompatibility of the membranes evaluated using the NIH-3T3 cell line. Data are presented as mean ± standard deviation.

In detail, in Figure 9A the cells appear sparsely distributed with limited adhesion on SF-Pgl; the cytoskeleton is poorly developed and the cells are isolated, indicating low affinity. In Figure 9B, the cells display improved adhesion on SF-Pgl/CI compared to SF-Pgl; they exhibit an elongated morphology with well-defined actin fibers, suggesting good cytoskeletal organization. Image analysis indicates that SF-Pgl/CI samples display a 20% increase in cellular coverage relative to SF-Pgl samples.

In Figure 9C, a high cell density is observed on SF-Pgl/VitB2; the nuclei are numerous, and the cells form an almost confluent layer with a uniform distribution of actin fibers. Figure 9D is similar to C, showing good proliferation and organization; the nuclei are regular, and the actin fibers form a continuous network, indicative of excellent cytocompatibility. Indeed, morphological analysis of the fluorescence images shows that 75% (±7%) of the sample is covered on SF-Pgl/VitB2 samples and 72% (±5%) on SF-Pgl/CI+VitB2 samples. The autofluorescence of the material prevents clear observation of the cell population. In addition, in Figure SI 4 we can observe the distribution of nuclei within the different samples. Overall, these observations indicate that the incorporation of CI enhances cell adhesion and cytoskeletal organization, whereas VitB2 promotes the formation of a well-organized, confluent cell layer, demonstrating the potential of SF-Pgl scaffolds functionalized with these bioactive compounds to support fibroblast proliferation and cytocompatibility.

### Adhesive-Free Tissue Bonding by Laser-Assisted Welding

All membranes naturally adhered to the various connective-tissue models tested, including tendons, corneas, and sclerae (Figure 10A,B,C). However, laser-assisted welding tests showed that mechanical resistance sufficient for handling was achieved only with membranes containing 1% CI (with or without VitB2), which provide a photothermal conversion rate of approximately 50% (Figure SI 5). This composition offers a favorable balance between energy efficiency and uniform heat distribution across the irradiated sample thickness. Adhesive-less bonding was obtained using optical pulses with a fluence of ∼ 0.42 J/cm^2^ or continuous-wave (CW) irradiation at a power density of ∼2–3 W/cm^2^ (Figure 10, upper panels). Although the exact values varied among tissues and across different testing locations, reflecting the natural biological variability and the inherent heterogeneity in local composition, hydration, and thickness, the overall trends remained consistent: lower power densities were generally insufficient to induce adhesion, whereas higher values resulted in thermal damage.

**Figure 10:**
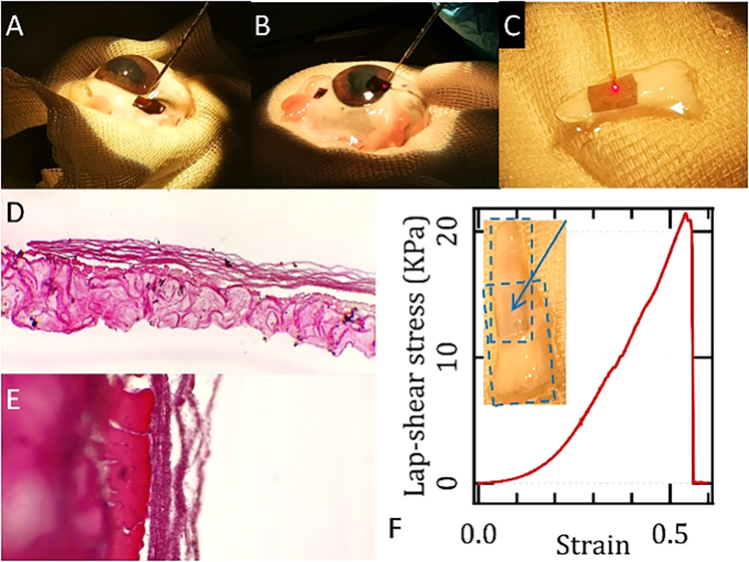
(A–C) Photographs show the experimental welding of SF-Pgl/CI+VitB2 membranes onto a lamb sclera (A), lamb cornea (B), and rabbit tendon (C). (D, E) Hematoxylin and eosin (H&E) stained images of the SF-Pgl/CI+VitB2 membrane welded to a rabbit tendon at 4× (D) and 20× (E) magnification. The membrane appears as a fiber bundle structure. The tendon shows no thermal damage, and the interface demonstrates clear adhesion. (F) Shear stress–strain curve of an SF-Pgl/CI+VitB2 membrane welded between two rabbit tendon stubs arranged in a sandwich configuration, suitable for lap-shear testing and simulating clinical injury repair. For this sample, the optical power was 500 mW CW, and the treated surface was ∼5 mm × 5 mm. The inset shows a photograph of the sample, with the arrow pointing to the film positioned between the two overlapping tendon stubs.

As an example of effective laser-welding performance, we simulated a tendon lesion in a rabbit model and applied CW irradiation at a power density of 2.88 W/cm^2^ (optical power: 500 mW; spot diameter: ∼2.3 mm). The lap-shear test (Figure 10F) showed that the welded membranes achieved an ultimate shear strength of approximately 20 kPa, comparable to materials commonly used for sealing tissue injuries and securing implants, such as commercially available fibrin sealants for cartilage repair.^42^ These results indicate that our membranes represent promising candidates for applications including tissue fixation and localized drug delivery.

The induced temperature during the welding process was maintained below 60 °C, which is within the optimal range for connective tissue welding (Figure SI 6). To verify the effectiveness of welding, histological analysis was performed using standard hematoxylin and eosin staining (Figure 10D,E). The results confirmed good adhesion at the membrane/tendon interface, with no evidence of thermal damage to the tissue. Based on investigations in similar systems using FTIR spectroscopy and molecular dynamics simulations at the interface between connective tissue and a polysaccharide film,^43^ we hypothesize that welding may occur through the disruption and successive formation of new intermolecular bonds at the membrane/tissue interface during heating and subsequent cooling.

## Conclusion

Silk fibroin-based nanofibrous membranes incorporating cuttlefish ink (1% w/w) and vitamin B2 (0.2% w/w) were successfully developed via water-based electrospinning. These membranes combine biocompatibility, biodegradability, and satisfactory mechanical performance, positioning them as promising scaffolds for soft tissue regeneration. CI exhibited notable antioxidant activity (EC50 = 200 μg), while the incorporation of both bioactive compounds enhanced SF β-sheet crystalline structure and slightly increased crystallization temperatures, as confirmed by ATR-FTIR and DSC analyses. VitB2 release followed Fickian diffusion, whereas CI remained retained within the matrix. NIH-3T3 fibroblast assays confirmed high cell viability (>80% at 48 h), with CI improving adhesion and cytoskeletal organization and VitB2 promoting formation of a confluent, well-organized cell layer. Laser-assisted welding demonstrated good adhesion to connective tissues, including tendons, corneas, and sclerae, with CI-containing membranes withstanding mechanical rupture at shear stresses of several kilopascals, while avoiding thermal damage (temperatures <60 °C), as confirmed by histological analysis. These results highlight that CI and VitB2 loaded fibroin membranes combine antioxidant, cytocompatible, and photothermal adhesive properties, making them promising scaffolds for tissue engineering, regenerative medicine, and controlled drug delivery. Ongoing studies using corneal epithelial cells and injured cornea models will further assess their applicability as next-generation scaffolds for critical ophthalmic applications.

## Experimental Section

### Materials

SF was extracted from *Bombyx mori* cocoons, purchased from CREA (Padova, Italy). The extraction was performed following a protocol reported in the literature^44^ and optimized at CNR-ISOF Bologna. Briefly, the cocoons were degummed in water by autoclaving at 120 °C for 1 h. The resulting SF fibers were rinsed with distilled water, dried, and subsequently dissolved in a 9.3 M LiBr solution at 60 °C for 6 h. The solution was then dialyzed against distilled water for 48 h using 12–14 kDa cellulose acetate dialysis membranes, and centrifuged to obtain pure regenerated SF solutions (approximately 6–7 wt/vol %). The final solution was stored at 4 °C. CI was extracted from the ink sacs of the common cuttlefish (Sepia officinalis) caught in the Adriatic Sea and purchased from a licensed seafood retailer (Fermo, Italy). The sacs were dissected using sterile disposable scalpels to isolate the ink. Approximately 10 g of CI were added to 50 mL of 1 M HCl and magnetically stirred for 3 h. After removal of undissolved solids, the suspension was stored overnight at 4 °C. It was then centrifuged (10,000 rpm, 10 min, 4 °C) and washed sequentially: three times with 1 M HCl, three times with acetone, and four times with Milli-Q water. The purified product was dispersed in 100 mL of Milli-Q water. The CI concentration, determined gravimetrically, was approximately 60 mg/mL.

VitB2 in form of powder was purchased from Farmalabor and used as an aqueous solution at a concentration of 0.20 mg/mL.

### Preparation of Electrospun Membranes of SF, CI, and VitB2

Electrospun nanofibrous membranes were prepared from aqueous solutions containing SF, glycerol (gl) (20% w/w vs SF), and poly­(ethylene oxide) (PEO (P), Mn 400 kDa, Sigma-Aldrich), with the addition of active compounds CI (1% w/w vs SF, representing the minimum quantity necessary for a consistent welding effect) and VitB2 (0.20% w/w vs SF), using an electrospinning system (Linari NanoTech Instrument). Based on previous studies we used 70:30 as the optimal SF:PEO ratio, with a total polymer concentration of 5% w/v.^45^ The following membrane formulations were prepared: SF-Pgl, SF-Pgl/CI, SF-Pgl/VitB2, and SF-Pgl/CI+VitB2. Solutions containing PEO, glycerol, and the active compounds were stirred overnight to allow complete solubilization of PEO, followed by the addition of SF solution. Electrospinning was conducted under the following conditions: 29–30 kV voltage, 0.01–0.02 mL/min flow rate, and a tip-to-collector distance of 19–20 cm. The collector was covered with aluminum foil to facilitate membrane removal.

### Characterization of Materials

#### DPPH Assay

The direct antioxidant activity of CI was evaluated with the 2,2-diphenyl-1-picrylhydrazyl (DPPH) assay, which relies on spectrophotometric measurements to determine the sample’s ability to scavenge free radicals. Accordingly, a 0.1 M DPPH solution was prepared in ethanol and increasing amounts of CI were allowed to react with 4 mL of the radical solution for 20 min. Following the reaction time, the UV–vis absorbance measurements at 516 nm were used to evaluate the inhibition percentage (eq 1) of the radical and the EC50.

1 $$I\%=\left[1-\frac{\left(A_{i}-A_{j}\right)}{A_{c}}\right]*100$$

*A_i_* = Absorbance of samples with CI and DPPH; *A_j_* = Absorbance of CI controls without DPPH; *A_c_* = Absorbance of DPPH solution without CI.

#### Optical, Thermal and Morphological Characterization

Spectroscopic analysis was performed using a PerkinElmer Lambda-650 UV–vis spectrophotometer. The infrared spectra were obtained using Attenuated Total Reflectance spectroscopy, employing a Bruker Vertex 70 interferometer equipped with a single reflection Platinum ATR accessory with diamond crystal. The measurements were conducted in the spectral region from 4000 to 400 cm^–1^, with 128 scans and a resolution of 4 cm^–1^. The curve fitting of overlapping bands of the infrared spectra covering the amide I and II regions (1480–1750 cm^–1^) was performed using the Levenberg–Marquardt algorithm implemented in OPUS 7.2. Quantitative analysis of the amide I and amide II bands was performed using curve fitting, second derivative and Fourier self-deconvolution methods, as already reported in literature.^46^ Spectra were processed using Bruker OPUS software. The amount of α-helices in a sample was computed as the ratio between the sum of the areas of the corresponding peaks in the amide I and amide II bands and the sum of the areas of all the peaks in the same spectral region. Thermal properties of membranes were analyzed using Differential Scanning Calorimetry (DSC, METTLER TOLEDO) under dry nitrogen flow (70 mL/min). Samples were heated from 35 to 300 °C at a rate of 5 °C/min. Morphological analysis of CI and electrospun membranes was carried out using a scanning electron microscope (SEM, Zeiss EVO LS 10 LaB6). Fiber and particle diameters (n = 100) were measured using ImageJ software (November 2024, National Institutes of Health (NIH)).

#### Stability and Biodegradability Tests

Membrane stability was assessed by immersing preweighed samples (30 ± 2 mg) in 2 mL of PBS (pH 7.4) and incubating them at 37 °C for 24 h. After incubation, the membranes were dried and reweighed to determine mass variation. Biodegradability was evaluated by calculating the percentage of mass loss after enzymatic degradation using a 0.5 mg/mL solution of protease XIV in PBS (pH 7.4). Samples were incubated at 37 °C, and degradation was assessed at 1, 3, 6, 24, and 48 h. At each time point, membranes were harvested, rinsed, dried at 50 °C, weighed, and subsequently reimmersed in fresh protease solution for continued digestion. All experiments were performed in triplicate.

#### Swelling Experiment

To conduct the swelling studies, SF-Pgl and SF-Pgl/CI electrospun membranes were first weighed in the dried state (*W*_D_) and then immersed in PBS at 37 °C for 24 h. The soaked membranes were carefully weighed in their swollen state (*W*_S_), and the swelling ratio (SD) was calculated using the following equation:

2 $$\mathrm{SD}=\frac{W_{s}-W_{D}}{W_{D}}$$

All experiments were performed in triplicate.

#### Young Modulus and Load Resistance

A custom-made testing device (AS Tessuti, Asper s.r.l., Italy) was employed to assess the elastic modulus and resistance to tensile load of the membranes. Each membrane was mounted using miniature clamps, and stress–strain curves were generated at a constant displacement rate of 50 μm/s until material failure.

#### Vitamin B2 Release

Preweighed membranes were immersed in 2 mL of PBS (pH 7.4), in order to ensure sink conditions and incubated at 37 °C. At predetermined time points (5, 15, 30 min; 1, 2, 3, and 6 h), aliquots were collected and analyzed by UV–Vis spectroscopy at λ = 445 nm. Quantification was performed using a calibration curve prepared with VitB2 solutions (0.005–0.05 mg/mL) in PBS. Control samples (SFgl) were also analyzed to account for potential background interference. All experiments were performed in triplicate. Release profiles were fitted using the Korsmeyer–Peppas and Peppas–Sahlin models to investigate the underlying release kinetics and mechanisms. A preliminary assessment of the release of VitB2 to a model tissue was also conducted by keeping membranes in contact with rabbit tendons harvested from food-grade animals in a climatic chamber with 37 °C, 100% RH, and then observed under a surgical microscope.

#### Biocompatibility Assay

Biocompatibility was assessed using NIH-3T3 murine fibroblasts, a well-established cell line used in tissue engineering studies. Cells were cultured in DMEM supplemented with 10% FBS, 1% Pen-Strep, 0.1 mM nonessential amino acids, and 2 mM l-glutamine. The Resazurin Reduction Assay (Sigma-Aldrich) was used to evaluate cell viability. Resazurin, a nontoxic blue dye, is reduced by metabolically active cells into resorufin, which is pink and fluorescent. Cells (50,000 cells/ml) were seeded onto the membranes and incubated for 24 and 48 h. After incubation, 10% resazurin solution was added and incubated for 4 h at 37 °C. Fluorescence was then measured using a plate reader (Thermo Scientific Varioskan Flash Multimode Reader). Analysis of cell adhesion was performed using ImageJ software (NIH).^47^

#### Laser-Assisted Welding Tests and Histological Analysis

Welding tests were performed on rabbit tendons and lamb corneas and sclerae harvested from food-grade animals and used within 4 h of sacrifice. All specimens were kept hydrated with physiological saline solution during testing. Two protocols were followed: (1) direct welding of membranes onto the tendon, cornea and sclera surface; (2) simulation of tendon injury by making a transverse cut followed by application and welding of the membrane. A near-infrared diode laser (810 nm, DEKA M.E.L.A. S.r.l., Italy) coupled to a 300 μm core diameter optical fiber (NA 0.22) was used. In protocol (1), the fiber tip was kept in contact with the specimens, and light was delivered with an optical power of 300 mW for 1 s, resulting in an energy density of 0.42 J/cm^2^. In protocol (2), the fiber tip was held 1 cm from the surface and moved continuously to avoid overheating (2.9 W/cm^2^ optical power). Laser power was adjusted from case to case, in order to determine the minimum effective dose for stable adhesion. Temperature during welding was monitored using an infrared thermal camera (NEC R300SR, Japan). The ultimate shear strength of the welded tendons was determined according to the lap-shear standard method ASTM F2255–24,^48^ following protocol (2), using the same device employed for material characterization (AS Tessuti, Asper s.r.l., Italy). In this application scenario, histological analysis was also performed using hematoxylin-eosin staining. Immediately after welding, samples were fixed in 4% paraformaldehyde for 16 h at room temperature, washed in PBS, embedded in Tissue-Tek matrix, and frozen at −20 °C. Sections (10 μm thick) were obtained using a cryostat (ThermoFisher Scientific, HM 525) and mounted on adhesive slides. Slides were stained with hematoxylin for 60 s and eosin for 15 s, washed, dried, and mounted with coverslips. Images were acquired using a Leica DM500 microscope equipped with a 5 MP Leica IC50 camera.

## Supplementary Material



## Notes

Fiber diameter distribution, Quantification of the secondary structure elements, UV–vis absorbance spectra of the release medium, Photograph of rabbit tendon, Fluorescence microscopy images, Absolute optical extinction spectra of hydrated SF-Pgl and SF-Pgl/CI samples, Thermograph and temperature time-course in a rabbit tendon during welding (PDF)

## References

[1] Ark M., Cosman P. H., Boughton P., Dunstan C. R. (2016). Review: Photochemical
Tissue Bonding (PTB) Methods for Sutureless Tissue Adhesion. Int. J. Adhes. Adhes..

[2] Chiulan I., Heggset E. B., Voicu ŞI., Chinga-Carrasco G. (2021). Photopolymerization
of Bio-Based Polymers in a Biomedical Engineering Perspective. Biomacromolecules.

[3] Chen Y., Wang K., Huang J., Li X., Rui Y. (2024). An Extensive
Evaluation of Laser Tissue Welding and Soldering Biotechnologies:
Recent Advancements, Progress, and Applications. Curr. Res. Biotechnol..

[4] Sagnella A., Pistone A., Bonetti S., Donnadio A., Saracino E., Nocchetti M., Dionigi C., Ruani G., Muccini M., Posati T., Benfenati V., Zamboni R. (2016). Effect of Different
Fabrication Methods on the Chemo-Physical Properties of Silk Fibroin
Films and on Their Interaction with Neural Cells. RSC Adv..

[5] Benfenati V., Toffanin S., Capelli R., Camassa L. M. A., Ferroni S., Kaplan D. L., Omenetto F. G., Muccini M., Zamboni R. (2010). A Silk Platform
That Enables Electrophysiology and Targeted Drug Delivery in Brain
Astroglial Cells. Biomaterials.

[6] Dionigi C., Posati T., Benfenati V., Sagnella A., Pistone A., Bonetti S., Ruani G., Dinelli F., Padeletti G., Zamboni R., Muccini M. (2014). A Nanostructured Conductive Bio-Composite
of Silk Fibroin-Single Walled Carbon Nanotubes. J. Mater. Chem. B.

[7] Ye J., Xie B., Hu J., Xu X., Lu S., Wang J., Yang L. (2025). Recent Advances in
Silk Fibroin-Based Biomaterials for Tissue Engineering
Applications. Int. J. Biol. Macromol..

[8] Eftekhari B. S., Ashtari B., Jahani M., Afjeh-Dana E., Janmey P. A., Simorgh S., Gholipourmalekabadi M. (2025). Silk Fibroin-Based
Matrices for the Guidance of Cell Interaction, Tissue Regeneration,
and Crosstalk. Macromol. Biosci..

[9] Harishchandra
Yadav R., Kenchegowda M., Angolkar M., T S M., Ali M Osmani R., Palaksha S., Veerabhadrappa Gangadharappa H. (2024). A Review of
Silk Fibroin-Based Drug Delivery Systems and Their Applications. Eur. Polym. J..

[10] Deng L., Hou M., Lv N., Zhou Q., Hua X., Hu X., Ge X., Zhu X., Xu Y., Yang H., Chen X., Liu H., He F. (2024). Melatonin-Encapsuled Silk Fibroin Electrospun Nanofibers
Promote Vascularized Bone Regeneration through Regulation of Osteogenesis-Angiogenesis
Coupling. Mater. Today Bio.

[11] Chen K., Li Y., Li Y., Pan W., Tan G. (2023). Silk Fibroin Combined
with Electrospinning as a Promising Strategy for Tissue Regeneration. Macromol. Biosci..

[12] Powers H. J. (2003). Riboflavin
(Vitamin B-2) and Health12. Am. J. Clin. Nutr..

[13] Pinto J. T., Zempleni J. (2016). Riboflavin. Adv. Nutr..

[14] Raiskup F., Spoerl E. (2013). Corneal Crosslinking with Riboflavin and Ultraviolet
A. I. Principles. Ocul. Surf..

[15] Raiskup F., Spoerl E. (2013). Corneal Crosslinking
with Riboflavin and Ultraviolet
A. Part II. Clinical Indications and Results. Ocul. Surf..

[16] Li L., Bao H., Zhang E., Wu S., Jiang X., Xiao Y., Fan S., Luo Y., Huang Y., Zhang P., Swain M., Elsheikh A., Chen S., Zheng X. (2025). Effect of Corneal Cross-Linking
on Biomechanical Properties of Swollen Rabbit Corneas. Exp. Eye Res..

[17] Lakshmi R., Lakshmi A. V., Bamji M. S. (1989). Skin Wound Healing
in Riboflavin
Deficiency. Biochem. Med. Metab. Biol..

[18] Cheung I. M. Y., McGhee C. N. J., Sherwin T. (2014). Beneficial Effect of the Antioxidant
Riboflavin on Gene Expression of Extracellular Matrix Elements, Antioxidants
and Oxidases in Keratoconic Stromal Cells. Clin.
Exp. Optom..

[19] Xu Z., Chen Z., Wang W., Meng X., Wang X., Xia Y., Meng Q., Li Y., Song R., Chen G. (2024). Cuttlefish
Ink-Derived Melanin Nanoparticle-Embedded Tremella Fuciformis Polysaccharide
Hydrogels for the Treatment of MRSA-Infected Diabetic Wounds. Int. J. Biol. Macromol..

[20] Menichetti A., Mordini D., Montalti M. (2024). Melanin as
a Photothermal Agent in
Antimicrobial Systems. Int. J. Mol. Sci..

[21] Nune M., Manchineella S., Govindaraju T., Narayan K. S. (2019). Melanin Incorporated
Electroactive and Antioxidant Silk Fibroin Nanofibrous Scaffolds for
Nerve Tissue Engineering. Mater. Sci. Eng.,
C.

[22] Mavridi-Printezi A., Menichetti A., Mordini D., Amorati R., Montalti M. (2023). Recent Applications
of Melanin-like Nanoparticles as Antioxidant Agents. Antioxidants.

[23] Roy S., Kim H. C., Kim J. W., Zhai L., Zhu Q. Y., Kim J. (2020). Incorporation of Melanin
Nanoparticles Improves UV-Shielding, Mechanical
and Antioxidant Properties of Cellulose Nanofiber Based Nanocomposite
Films. Mater. Today Commun..

[24] Posati T., Ferroni C., Aluigi A., Guerrini A., Rossi F., Tatini F., Ratto F., Marras E., Gariboldi M. B., Sagnella A., Ruani G., Zamboni R., Varchi G., Sotgiu G. (2018). Mild and Effective
Polymerization of Dopamine on Keratin
Films for Innovative Photoactivable and Biocompatible Coated Materials. Macromol. Mater. Eng..

[25] Ryabkin D., Meglinsk I., Gerasimenko A. (2023). Amendments
of Weld Formation in Human
Skin Laser Soldering. J. Biophotonics.

[26] Chen S., Cai D., Dong Q., Ma G., Xu C., Bao X., Yuan W., Wu B., Fang B. (2023). Silver Nanoparticles–Decorated
Extracellular Matrix Graft: Fabrication and Tendon Reconstruction
Performance. Biomater. Res..

[27] Ratto F., Matteini P., Rossi F., Menabuoni L., Tiwari N., Kulkarni S. K., Pini R. (2009). Photothermal
Effects
in Connective Tissues Mediated by Laser-Activated Gold Nanorods. Nanomedicine.

[28] Dong J., Breitenborn H., Piccoli R., Besteiro L. V., You P., Caraffini D., Wang Z. M., Govorov A. O., Naccache R., Vetrone F., Razzari L., Morandotti R. (2020). Terahertz
Three-Dimensional Monitoring of Nanoparticle-Assisted Laser Tissue
Soldering. Biomed. Opt. Express.

[29] Derby C. D. (2014). Cephalopod
Ink: Production, Chemistry, Functions and Applications. Mar. Drugs.

[30] de
la Calle I., Soto-Gómez D., Pérez-Rodríguez P., López-Periago J. E. (2019). Particle Size Characterization of
Sepia Ink Eumelanin Biopolymers by SEM, DLS, and AF4-MALLS: A Comparative
Study. Food Anal. Methods.

[31] Ju K.-Y., Lee Y., Lee S., Park S. B., Lee J.-K. (2011). Bioinspired Polymerization
of Dopamine to Generate Melanin-Like Nanoparticles Having an Excellent
Free-Radical-Scavenging Property. Biomacromolecules.

[32] Barbalinardo M., Giannelli M., Forcini L., Luppi B., Donnadio A., Navacchia M. L., Ruani G., Sotgiu G., Aluigi A., Zamboni R., Posati T. (2022). Eco-Sustainable Silk Fibroin/Pomegranate
Peel Extract Film as an Innovative Green Material for Skin Repair. Int. J. Mol. Sci..

[33] Insińska-Rak M., Golczak A., Sikorski M. (2012). Photochemistry of Riboflavin Derivatives
in Methanolic Solutions. J. Phys. Chem. A.

[34] Lu Q., Zhang B., Li M., Zuo B., Kaplan D. L., Huang Y., Zhu H. (2011). Degradation Mechanism
and Control
of Silk Fibroin. Biomacromolecules.

[35] Hu X., Kaplan D., Cebe P. (2008). Dynamic Protein–Water
Relationships
during β-Sheet Formation. Macromolecules.

[36] Lu S., Wang X., Lu Q., Zhang X., Kluge J. A., Uppal N., Omenetto F., Kaplan D. L. (2010). Insoluble and Flexible
Silk Films Containing Glycerol. Biomacromolecules.

[37] Lee J., Choi H. N., Cha H. J., Yang Y. J. (2023). Microporous Hemostatic
Sponge Based on Silk Fibroin and Starch with Increased Structural
Retentivity for Contact Activation of the Coagulation Cascade. Biomacromolecules.

[38] Yang L., Wang X., Xiong M., Liu X., Luo S., Luo J., Wang Y. (2024). Electrospun Silk Fibroin/Fibrin
Vascular Scaffold with
Superior Mechanical Properties and Biocompatibility for Applications
in Tissue Engineering. Sci. Rep..

[39] Xiang J., Li Y., Ren M., He P., Liu F., Jing Z., Li Y., Zhang H., Ji P., Yang S. (2022). Sandwich-like Nanocomposite
Electrospun Silk Fibroin Membrane to Promote Osteogenesis and Antibacterial
Activities. Appl. Mater. Today.

[40] Miguel S. P., Simões D., Moreira A. F., Sequeira R. S., Correia I. J. (2019). Production
and Characterization of Electrospun Silk Fibroin Based Asymmetric
Membranes for Wound Dressing Applications. Int.
J. Biol. Macromol..

[41] Alkazemi H., Chai J., Allardyce B. J., Lokmic-Tomkins Z., O’Connor A. J., Heath D. E. (2025). Glycerol-Plasticized
Silk Fibroin
Vascular Grafts Mimic Key Mechanical Properties of Native Blood Vessels. J. Biomed. Mater. Res. Part A.

[42] Amirhekmat A., Brown W. E., Salinas E. Y., Hu J. C., Athanasiou K. A., Wang D. (2024). Mechanical Evaluation
of Commercially Available Fibrin Sealants for
Cartilage Repair. Cartilage.

[43] Matteini P., Ratto F., Rossi F., Centi S., Dei L., Pini R. (2010). Chitosan Films Doped
with Gold Nanorods as Laser-Activatable Hybrid
Bioadhesives. Adv. Mater..

[44] Rockwood D. N., Preda R. C., Yücel T., Wang X., Lovett M. L., Kaplan D. L. (2011). Materials Fabrication
from Bombyx Mori Silk Fibroin. Nat. Protoc..

[45] Chutipakdeevong J., Ruktanonchai U. R., Supaphol P. (2013). Process Optimization of Electrospun
Silk Fibroin Fiber Mat for Accelerated Wound Healing. J. Appl. Polym. Sci..

[46] Hu X., Kaplan D., Cebe P. (2006). Determining
Beta-Sheet Crystallinity
in Fibrous Proteins by Thermal Analysis and Infrared Spectroscopy. Macromolecules.

[47] Schneider C. A., Rasband W. S., Eliceiri K. W. (2012). NIH Image
to ImageJ: 25 Years of
Image Analysis. Nat. Methods.

[48] International, A. Standard Test Method for Strength Properties of Tissue Adhesives in Lap-Shear by Tension Loading ASTM Int. 2024.

