# Supplementary material for: Sustainable Silk Fibroin Nanofibers Membranes with Natural Photothermal and Bioactive Components for Adhesive-Free Soft Tissue Repair
Source: ACS Appl Bio Mater. 2026 Jan 23;9(4):2047–57. doi: 10.1021/acsabm.5c02042 (PMC12914641; doi:10.1021/acsabm.5c02042)
Supplement: Supplementary file 1 [file mt5c02042_si_001.pdf]

Supporting Information

Sustainable Silk Fibroin Nanofibers Membranes with Natural Photothermal and Bioactive Components for Adhesive-Free Soft Tissue Repair

*Martina Corsini[±1], Livia Ottaviano[±1], Luana Mariani[±1], Marianna Barbalinardo[#], Giada Magni[ϕ], Francesca Rossi[ϕ], Fulvio Ratto[ϕ], Anna Donnadio[§], Roberto Zamboni[±], Annalisa Aluigi[¥], Giovanna Sotgiu[±] and Tamara Posati[±]**

± Consiglio Nazionale delle Ricerche, Istituto per la Sintesi Organica e la Fotoreattività (CNR-ISOF), via Piero Gobetti 101, 40129 Bologna, Italy.

Consiglio Nazionale delle Ricerche, Istituto per lo Studio dei Materiali Nanostrutturati (CNR-ISMN), via Piero Gobetti 101, 40129 Bologna, Italy.

ϕ Consiglio Nazionale delle Ricerche Istituto di Fisica Applicata Nello Carrara (CNR-IFAC) Via Madonna del Piano 10, 50019 Sesto Fiorentino, Italy.

§ Dipartimento di Scienze Farmaceutiche, Università di Perugia, Via del Liceo, 1, 06123 Perugia, Italy.

¥ Dipartimento di Scienze Biomolecolari, Università di Urbino, Piazza del Rinascimento 6, 61029 Urbino, Italy.

1 These authors equally contributed to this work.

* corresponding author

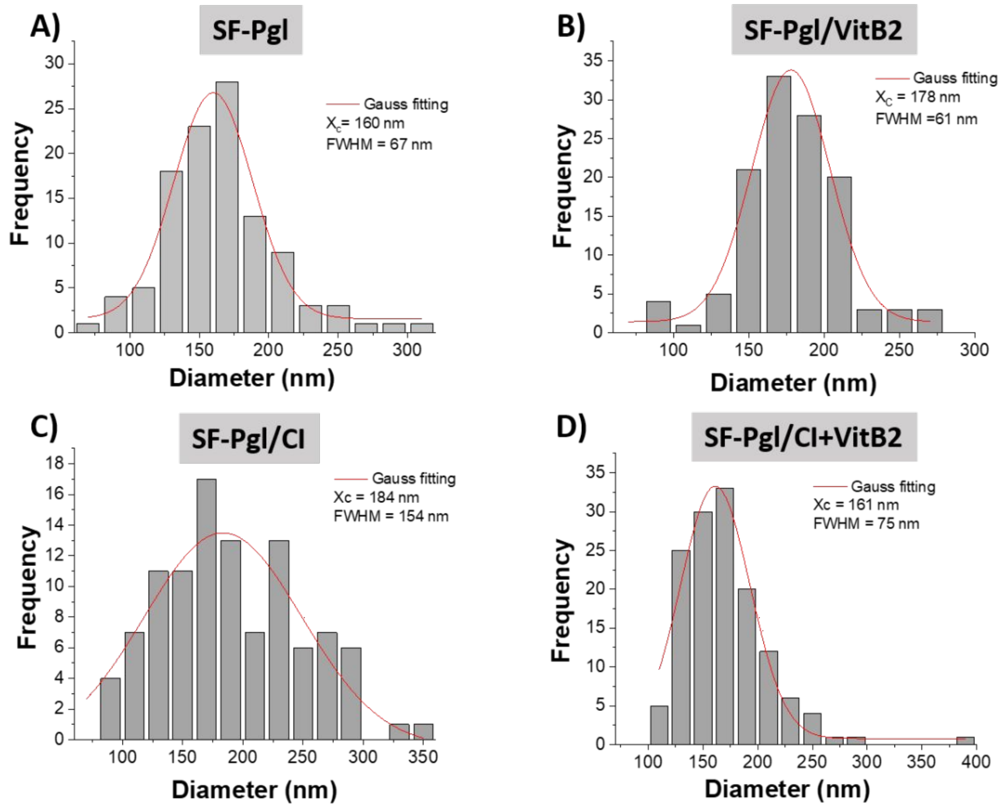

Figure S1. Fiber diameter distribution of A) SF-Pgl, B) SF-Pgl/VitB2, C) SF-Pgl/CI and D) SF-Pgl/CI+VitB2.

Table S1. Quantification of the secondary structure elements on the SF membranes.

**(cm^{-1})**	**Area SF-Pgl**	**Area SF-Pgl/VitB2**	**Area SF-Pgl/CI**	**Area SF-Pgl/CI+VitB2**	**Assignment**
1515	5.21	14.38	9.57	11.28	β-structures
1545	17.02	5.40	7.07	6.43	α-helix/random coil; VitB2
1620	5.37	3.76	4.86	6.51	β-structures
1645	24.35	16.80	11.42	10.38	α-helix/random coil; VitB2
1700	1.75	0.47	0.51	0.88	β-structures

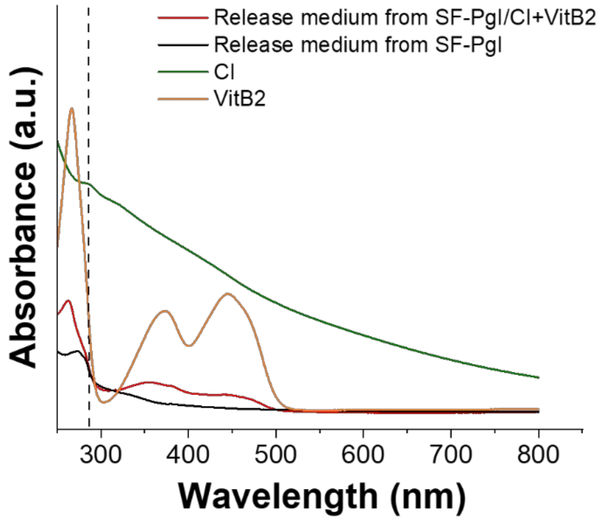

Figure S2. UV–Vis absorbance spectra of the release medium collected from SF-Pgl fibers (blank control, black line), the release medium collected from SF-Pgl/CI+VitB2 fibers after 24 h (red line), CI standard dispersion (olive line) and VitB2 standard solution (orange line). Only the characteristic peaks of VitB2 are detected in the release medium. The broad absorption/scattering profile typical of CI nanoparticles is absent, confirming that no detectable CI is released from the fibers.

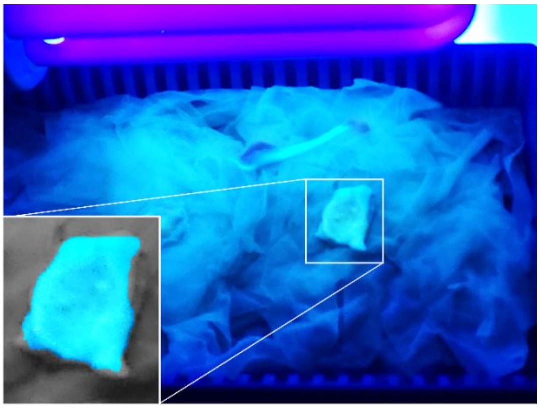

Figure S3. Photograph of a rabbit tendon, shown after 5 hours of incubation with an SF-Pgl/CI+VitB2 membrane in a humidified chamber at 37°C. The image was then acquired under UV light exposure.

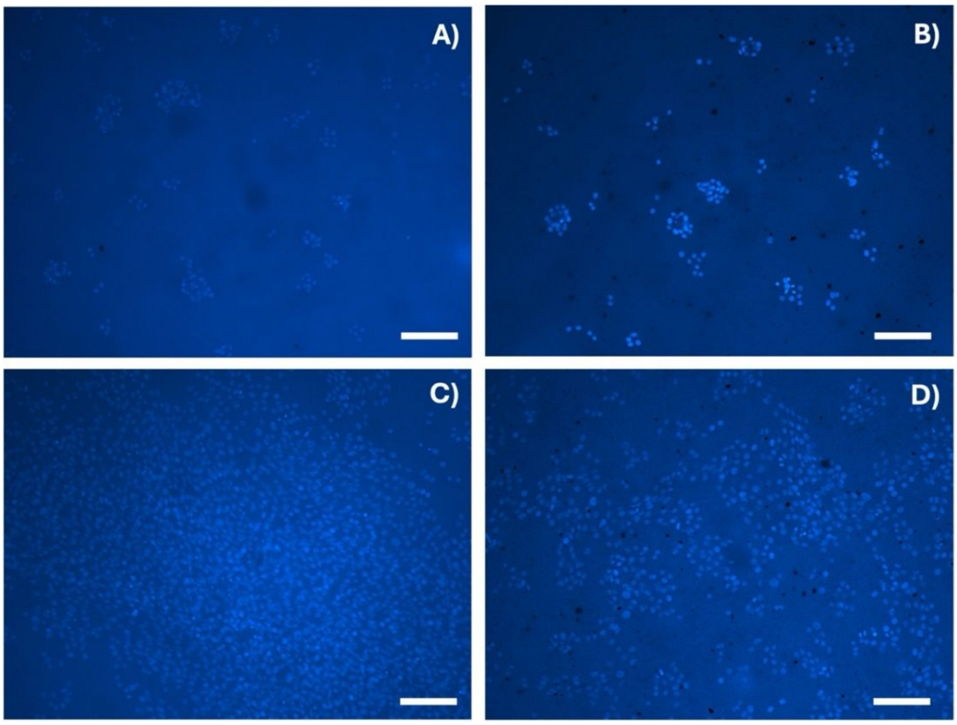

Figure S4. Fluorescence microscopy images of fibroblasts (NIH-3T3) cultured on A) SF-Pgl, B) SF-Pgl/CI, C) SF-Pgl/VitB2, and D) SF-Pgl/CI+VitB2. Cells were stained for nuclei (blue) using DAPI. Scale bar: 100 µm.

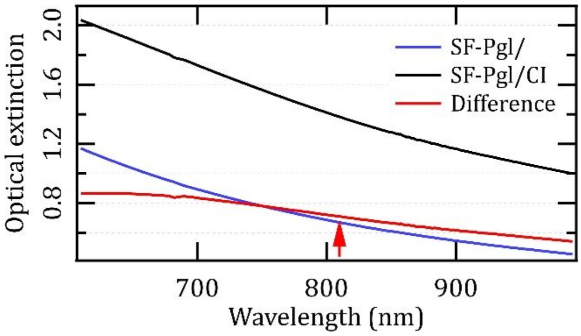

Figure S5. Absolute optical-extinction spectra for 80 µm-thick hydrated SF-Pgl and SF-Pgl/CI samples, together with their difference, which is taken to represent the contribution of melanin particles to absorption and scattering. The emission line of the diode laser used in the welding experiments is indicated by an arrow. To estimate the overall optical absorption of the membranes at the laser wavelength, we compared the extinction of hydrated SF-Pgl/CI samples with that of SF-Pgl

controls of identical thickness. In this analysis, we conservatively assumed that the control membranes contribute exclusively to scattering and that the extinction of *Sepia officinalis* eumelanin consists of approximately equal contributions from scattering and absorption [1]. Under these assumptions, the estimated absorption at 810 nm was about 0.35, corresponding to a photothermal conversion efficiency of roughly 55%.

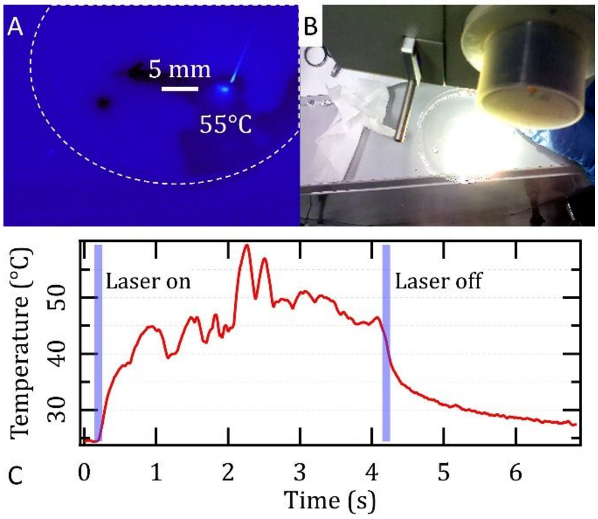

Figure S6. A) False-color thermal-camera image of a rabbit tendon during welding, showing that heating remained confined to a spot of approximately 1 mm in diameter. B) Corresponding photograph of the setup. C) Time evolution of the maximum temperature within the irradiated region, indicating that, in this example, the localized heating consistently remained below 60 °C.

(1) Bashkatov, A. N.; Genina, E. A.; Kochubey, V. I.; Stolnitz, M. M.; Bashkatova, T. A.; Novikova, O. V; Peshkova, A. Y.; Tuchin, V. V. Optical Properties of Melanin in the Skin and Skinlike Phantoms. In *Proc.SPIE*; 2000; Vol. 4162, pp 219–226. https://doi.org/10.1117/12.405946.
